# Electrophysiological Assessment in Birdshot Chorioretinopathy: Flicker Electroretinograms Recorded With a Handheld Device

**DOI:** 10.1167/tvst.11.5.23

**Published:** 2022-05-20

**Authors:** Anna M. Waldie, Angharad E. Hobby, Isabelle Chow, Elisa E. Cornish, Mathura Indusegaran, Aleksandra Pekacka, Phuc Nguyen, Clare Fraser, Alison M. Binns, Miles R. Stanford, Christopher J. Hammond, Peter J. McCluskey, John R. Grigg, Omar A. Mahroo

**Affiliations:** 1 Save Sight Institute, Faculty of Medicine and Health, The University of Sydney, Sydney, New South Wales, Australia; 2 The Speciality of Ophthalmology Faculty of Medicine and Health University of Sydney, Camperdown, New South Wales, Australia; 3 Section of Ophthalmology, King's College London, London, UK; 4 Department of Ophthalmology, Guy's and St Thomas’ Hospital NHS Foundation Trust, London, UK; 5 Division of Optometry and Visual Sciences, City, University of London, London, UK; 6 NIHR Biomedical Research Centre at Moorfields Eye Hospital and UCL Institute of Ophthalmology, London, UK; 7 Institute of Ophthalmology, University College London, London, UK

**Keywords:** birdshot chorioretinopathy, electroretinography, retina, uveitis

## Abstract

**Purpose:**

The flicker electroretinogram (ERG) is a sensitive indicator of retinal dysfunction in birdshot chorioretinopathy (BCR). We explored recordings from a handheld device in BCR, comparing these with conventional recordings in the same patients and with handheld ERGs from healthy individuals.

**Methods:**

Non-mydriatic flicker ERGs, using the handheld RETeval system (LKC Technologies), were recorded with skin electrodes at two centers. At one center (group 1), the stimuli (85 Td·s, 850 Td background) delivered retinal illuminance equivalent to international standards; at the other center (group 2), a different protocol was used (32 Td·s, no background). Patients also underwent international standard flicker ERG recordings with conventional electrodes following mydriasis. Portable ERGs from patients were also compared with those from healthy individuals.

**Results:**

Thirty-two patients with BCR (mean age ± SD, 56.4 ± 11.3 years) underwent recordings. Portable and standard ERG parameters correlated strongly (*r* > 0.75, *P* < 0.01) in both groups. Limits of agreement for peak times were tighter in group 1 (*n* = 21; −4.3 to +2.0 ms [right eyes], −3.9 to 1.5 ms [left eyes]) than in group 2 (*n* = 11; −3.4 to +6.9 ms [right eyes], −4.8 to +9.0 ms [left eyes]). Compared with healthy controls (*n* = 66 and *n* = 90 for groups 1 and 2, respectively), patients with BCR showed smaller mean amplitudes and longer peak times.

**Conclusions:**

Portable ERGs correlated strongly with conventional recordings, suggesting potential in rapid assessment of cone system function in office settings.

**Translational Relevance:**

Flicker ERGs, known to be useful in BCR, can be obtained rapidly with a portable device with skin electrodes and natural pupils.

## Introduction

Birdshot chorioretinopathy (BCR) is a rare form of chronic, bilateral, posterior uveitis with a distinctive phenotype and a strong association with HLA-A29. Ryan and Maumenee^1^ coined the term “birdshot retinochoroidopathy” in 1980 in a case series of 13 patients with a disease process characterized by a white, painless eye with minimal anterior segment inflammation but particulate vitreous debris, with profuse retinal vascular leakage and resultant retinal, macular, and disc oedema. Patients had multiple pale fundal lesions, and the authors noted a resemblance between these and the “pattern seen with birdshot in the scatter from a shotgun.”^1^ Patients often present in middle age with a range of visual symptoms including blurred vision, floaters, nyctalopia, and dyschromatopsia. Given the rarity of this condition, diagnosis and treatment are frequently delayed, risking significant visual loss.

Assessing disease activity in BCR can be challenging. Previous studies have suggested that visual electrophysiological testing, particularly the electroretinogram (ERG), may detect early changes and could be useful to monitor disease progression in BCR and other retinal inflammatory diseases.^2^^–^^5^ The international standard^6^ light-adapted full-field 30-Hz flicker ERG peak time has been consistently noted as the most sensitive parameter for retinal dysfunction in BCR (a delayed peak time indicating retinal dysfunction) and can be used to monitor progress and evaluate treatment efficacy, thus helping to guide therapeutic decisions.^2^^,^^4^^,^^5^

Standard full-field ERG testing requires pharmacological pupil dilation, uses corneal electrodes, and entails a number of dark-adapted and light-adapted recordings that may take around an hour to complete.^6^^,^^7^ It usually takes place in specialist centers, often requiring patients to undergo additional hospital visits. The RETeval (LKC Technologies, Gaithersburg, MD) is a handheld, mydriasis-free, full-field ERG recording device that uses specialized skin electrodes to record ERGs.^8^^–^^12^ The device was originally developed to aid in diabetic retinopathy screening,^9^^,^^10^ and it incorporates an ∼30-Hz stimulus delivered in the absence of a background. Advanced versions of the device also deliver stimuli more similar to international standards, including the standard 30-Hz light-adapted stimulus (which is a white stimulus delivered on a specified standard white background). In this study, we explored the use of the portable device to obtain rapid recordings in the clinic setting in patients with BCR and compared these recordings with conventional recordings, as well as with portable recordings made in healthy individuals.

## Methods

### Ethical Approval

Informed consent was obtained from participants after explanation of the nature and possible consequences of the study. The research was conducted in accordance with the tenets of the Declaration of Helsinki and was approved by the United Kingdom's National Health Service Health Research Authority Research Ethics Service Committee (London – Harrow, 11/LO/2029) and the South Eastern Sydney Local Health District Human Research Ethics Committee (18/235 REGIS 2018/ETH00441), based in Australia.

### Study Centers

The study was prospective, and recruitment and recording from patients with BCR took place at two centers—namely, the Eye Department of St Thomas’ Hospital in London and the Save Sight Institute in Sydney, Australia. Healthy control participants were from the TwinsUK cohort,^13^ comprised of adult twins who have volunteered for research studies at St. Thomas’ Hospital.

### Portable ERG Recording

The RETeval system is a portable device that has been previously described.^8^^–^^12^ It has a camera and video display that allow live viewing of the participant's eye during recordings. A built-in pupilometer allows use with natural pupils; pupil diameter is monitored, and the stimulus strength can be adjusted to achieve the required retinal illuminance. Recordings were made using specialized skin electrodes (Sensor Strip; LKC Technologies) positioned over the inferior orbital rim. The strips contain an electrode array with three electrodes—an active (positive) electrode, a reference (negative) electrode, and a ground electrode—and sampling was at a rate of approximately 2 kHz.

Patients in London (group 1) underwent recordings elicited by the stimulus equivalent to the standard light-adapted flicker ERG. For this, the device delivers white 85-Td·s flashes at 28.3 Hz in the presence of an 850-Td white background; this is equivalent to the illuminance delivered by the International Society for Clinical Electrophysiology of Vision (ISCEV) standard stimulus of 3 cd·s/m^2^ on a 30-cd/m^2^ background through a 6-mm-diameter pupil. Recording time for each stimulus repetition ranged from 5 to 15 seconds depending on the result reliability. Patients in Sydney (group 2) underwent recordings to 32 Td·s flashes delivered at 28.3 Hz in the absence of a background; this latter stimulus protocol is more widely available with this device and was the protocol available at this center.

### Conventional ERG Recording

Conventional ERGs were recorded from all participating patients with BCR using the Diagnosys ColorDome system running Espion software (Diagnosys LCC, Lowell, MA), following pharmacological mydriasis, using the standard light-adapted 30-Hz flicker stimulus (3 cd·s/m^2^ on a 30-cd/m^2^ white background with sampling at 2 kHz). These were recorded using conventional recording electrodes. For patients in London, these were conductive fiber electrodes placed in the lower conjunctival fornix; in Sydney, gold foil electrodes were used. The indifferent electrode was placed at the temple and a ground electrode was positioned on the forehead; these were skin surface electrodes positioned following skin cleaning with alcohol wipes.

### Analyses

ERGs recorded from the right and left eyes of each participant were included; however, as both eyes of each participant are correlated, we conducted separate, independent analyses for right eyes and left eyes. The Kolmogorov–Smirnov test was used to test for deviation from a normal distribution. Pearson's correlation coefficient was calculated to evaluate the degree of correlation between the portable and conventionally recorded ERGs for both amplitudes and implicit times. Paired *t*-tests were used to evaluate differences between portable and conventional recordings. As the stimulus protocols for portable ERGs were different in the two centers, separate analyses were performed for patients recruited in London and Sydney.

Bland–Altman analyses were performed to quantify the agreement between values recorded from the handheld and conventional devices. As it was already expected that amplitudes would be significantly lower with the portable device (due to the use of skin electrodes), this analysis was performed only for peak times. The systematic mean difference was termed “bias.” Limits of agreement were calculated as the mean difference ± 1.96 SD, which provided an interval within which 95% of the difference between measurements by the two devices were expected to lie.

Finally, portable recordings in patients with BCR were also compared with those obtained in healthy controls for the two flicker ERG protocols (unpaired *t*-tests). For the device to be clinically useful, we would expect that patients with BCR on average should have longer ERG peak times and lower flicker ERG amplitudes compared with healthy individuals.

## Results

### Patient Demographics

Thirty-two patients (23 females and nine males) with clinical diagnoses of BCR were included. Mean age ± SD was 56.4 ± 11.3 years. Ages ranged from 28 to 72 years (median age, 58.5 years). Twenty-one patients (15 females and six males) were in group 1 and had a mean age ± SD of 55.8 ± 12.5 years (median age, 58 years). In group 1, the flicker ERG peak time with the portable device was not measurable in the right eye in one patient and in the left eye in another patient; in both cases, the response was of very low amplitude. Eleven patients (eight females and three males) were in group 2, with a mean age ± SD of 57.5 ± 8.9 years (median age, 60 years). All patients in group 2 had measurable flicker ERG amplitudes and peak times.

Mean time ± SD between diagnosis and the ERG recordings of the present study was 7.6 ± 5.7 years for the whole cohort. For group 1, the mean time ± SD since diagnosis was 9.7 ± 5.8 years, ranging from 1.8 to 23.8 years. For group 2, the mean time ± SD since diagnosis was 3.8 ± 3.0 years, ranging from 2 months to 8.7 years. Mean time since diagnosis was significantly longer for group 1 than group 2 (*P =* 0.0036). The majority of patients were on treatment (oral prednisolone and/or other immunosuppressive medication) at the time of the ERG recordings.

### Mean Values and Correlation Between Portable and Conventional ERGs

In group 1, mean ± SD peak times and amplitudes recorded with the handheld device were 30.2 ± 3.5 ms and 17.2 ± 11.3 µV, respectively, for right eyes, and 30.3 ± 4.0 ms and 15.7 ± 9.8 µV, respectively, for left eyes. For conventional recordings, corresponding values were 31.1 ± 3.9 ms and 47.8 ± 33.3 µV, respectively, for right eyes, and 31.8 ± 4.0 ms and 49.9 ± 29.2 µV, respectively, for left eyes. In group 2, mean ± SD peak times and amplitudes recorded with the handheld device were 34.1 ± 4.4 ms and 16.8 ± 8.4 µV, respectively, for right eyes, and 33.9 ± 5.1 ms and 20.4 ± 11.0 µV, respectively, for left eyes. For conventional recordings, corresponding values were 32.4 ± 4.1 ms and 80.2 ± 39.9 µV, respectively, for right eyes, and 31.8 ± 4.7 ms and 87.2 ± 41.4 µV, respectively, for left eyes. Kolmogorov–Smirnov testing did not detect significant deviation from a normal distribution.

**Figure 1.**
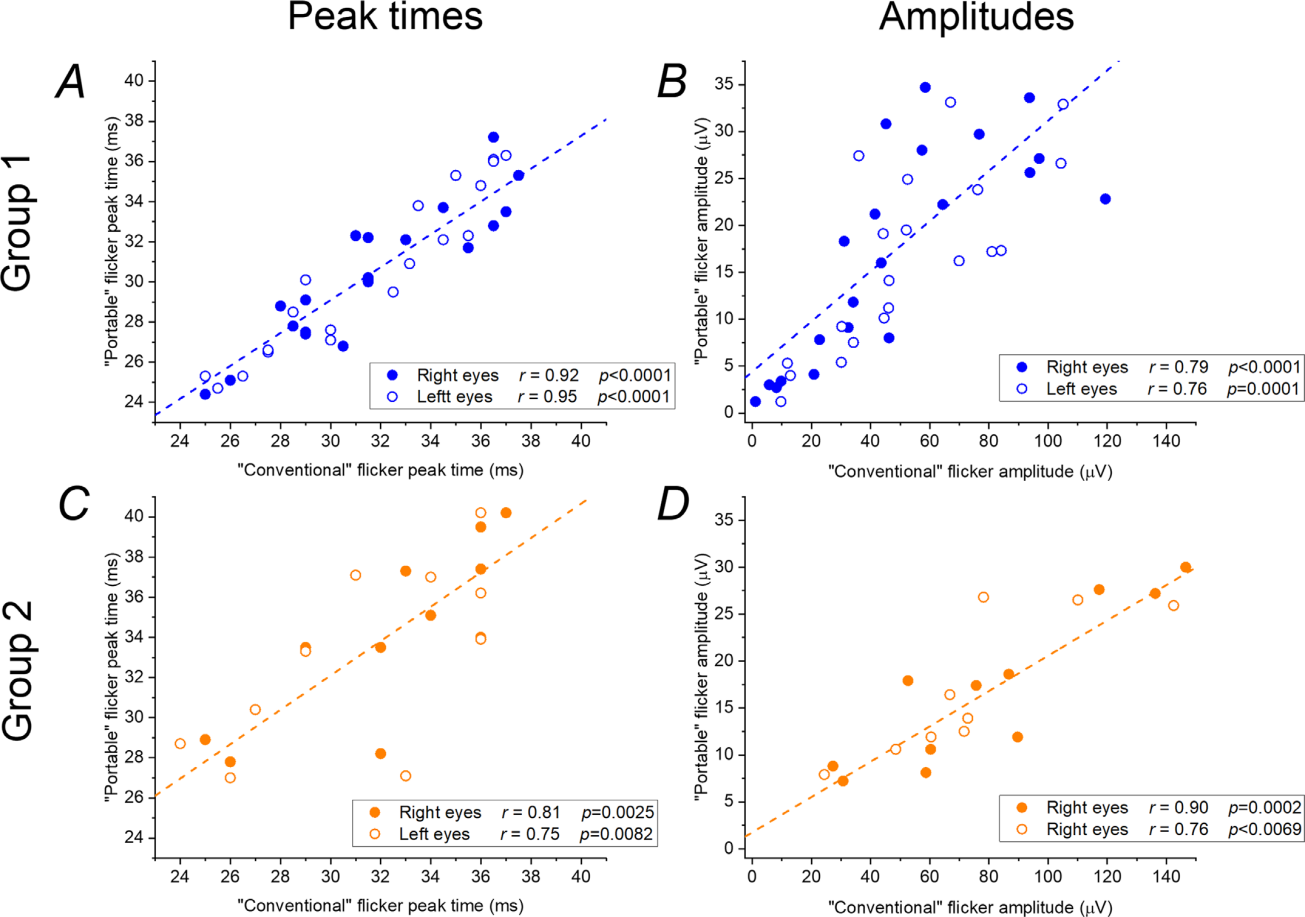
Flicker ERG parameters recorded with portable device and conventional methods in patients in group 1 (A, B) and group 2 (C, D). Peak times (A) and amplitudes (B) of ERGs recorded with skin electrodes in response to 28.3-Hz white flashes delivering 85 Td·s on an 850-Td white background using the portable system are plotted against parameters from conventionally recorded standard light-adapted 30-Hz flicker ERGs (recorded with conductive fiber electrodes). Peak times (C) and amplitudes (D) of ERGs recorded with skin electrodes in response to 28.3-Hz white flashes delivering 32 Td·s (with no background) using the portable system are plotted against parameters from conventionally recorded standard light-adapted 30-Hz flicker ERGs (recorded with gold foil electrodes). *Dashed lines* show simple linear trendlines fitted to the data for right eyes; these lines also provide a good fit to data for left eyes.

Figure 1 plots the portable ERG parameters against conventionally recorded parameters for the two groups, giving correlation coefficients. All panels show strong, very significant, correlations. The strongest correlation was for peak times in group 1, with coefficients of 0.92 (*P* < 0.0001) and 0.94 (*P* < 0.0001) for right and left eyes, respectively. As expected, amplitudes from skin electrode recordings (*y*-axis values in Figs. 1B and 1D) are markedly lower than those obtained with conventional electrodes (*x*-axis values in Figs. 1B and 1D). For group 1, conventionally recorded ERG amplitudes (with conductive fiber electrodes) were on average around 3 to 3.5 times higher than those recorded with the portable device with skin electrodes. The mean ± SD ratios were 2.9 ± 1.3 for right eyes and 3.6 ± 1.5 for left eyes; the median ratios were 2.9 and 3.3, respectively. For group 2 patients (whose conventional ERGs were recorded with gold foil electrodes), mean ± SD ratios of amplitudes were 4.9 ± 1.5 for right eyes and 4.5 ± 1.2 for left eyes; the median ratios were 4.7 and 4.6, respectively. Gold foil electrodes can give higher amplitudes than conductive fiber electrodes, and this may explain the higher ratios in group 2 and the shallower gradient of the regression line in Figure 1D compared with Figure 1B. Also, the stimulus delivered by the portable device in group 2 was less strong (32 Td·s compared with 85 Td·s in group 1).

Significant correlations between ERG parameters and time since diagnosis were found in group 1. There were negative correlations between amplitudes and time since diagnosis. For right eyes, the correlation coefficients were −0.75 (*P =* 0.00011) and −0.74 (*P =* 0.00014) for conventional and portable ERGs, respectively; for left eyes, they were −0.62 (*P =* 0.0027) and −0.62 (*P* = 0.0027), respectively. Positive correlations were found between peak times and time since diagnosis. For right eyes, coefficients were 0.46 (*P =* 0.0374) and 0.66 (*P =* 0.0016) for conventional and portable ERGs, respectively; for left eyes, they were 0.31 (*P =* 0.179) and 0.40 (*P =* 0.077), respectively. No statistically significant correlations with time since diagnosis were found in group 2.

### Agreement Between Recording Methods

**Figure 2.**
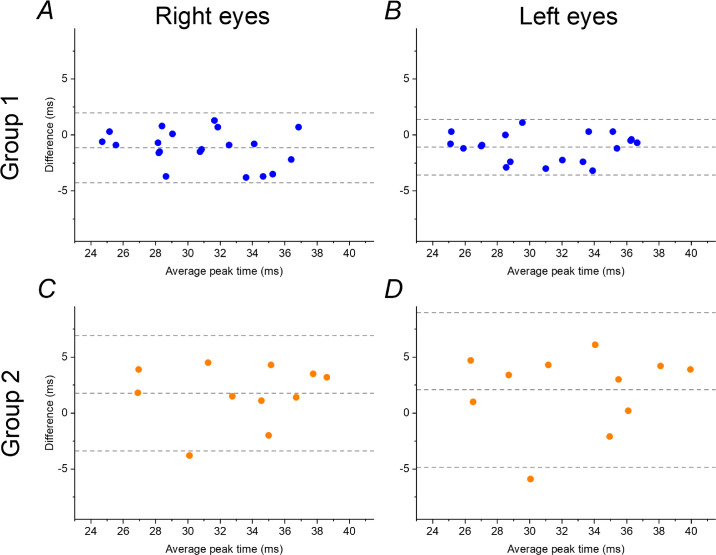
Bland–Altman plots of differences between peak times (portable minus conventionally recorded ERG) against average for group 1 (A, B) and group 2 (C, D). Stimulus parameters and recording methods are given in the text and in the legend to Figure 1. *Dashed lines* show mean difference and limits of agreement.

Amplitudes were significantly lower in both groups for portable recordings compared with conventional recordings (*P* < 0.0001). Peak times showed a very small difference. For group 1, mean peak times were significantly shorter with the portable device, with a mean difference of 1.1 ms (*P =* 0.0047) for right eyes and 1.2 ms (*P =* 0.0007) for left eyes. For group 2, portable ERG peak times were on average 1.8 ms and 2.1 ms longer than those for conventional ERGs for right and left eyes, respectively, but the difference did not quite reach significance (*P =* 0.0506 and *P =* 0.0797 for right and left eyes, respectively). Figure 2 shows Bland–Altman plots for peak times for the two groups. Mean differences (portable minus conventionally recorded ERGs) are plotted against average peak times (average of portable and conventionally recorded ERG peak times). The upper panels (group 1) show tighter agreement, with limits of agreement of −4.3 and +2.0 ms for right eyes and −3.9 and +1.5 ms for left eyes. For group 2, the limits of agreement were −3.4 and +6.9 ms for right eyes and −4.8 and 9.0 ms for left eyes.

### Comparison with Portable Recordings in Healthy Individuals

**Figure 3.**
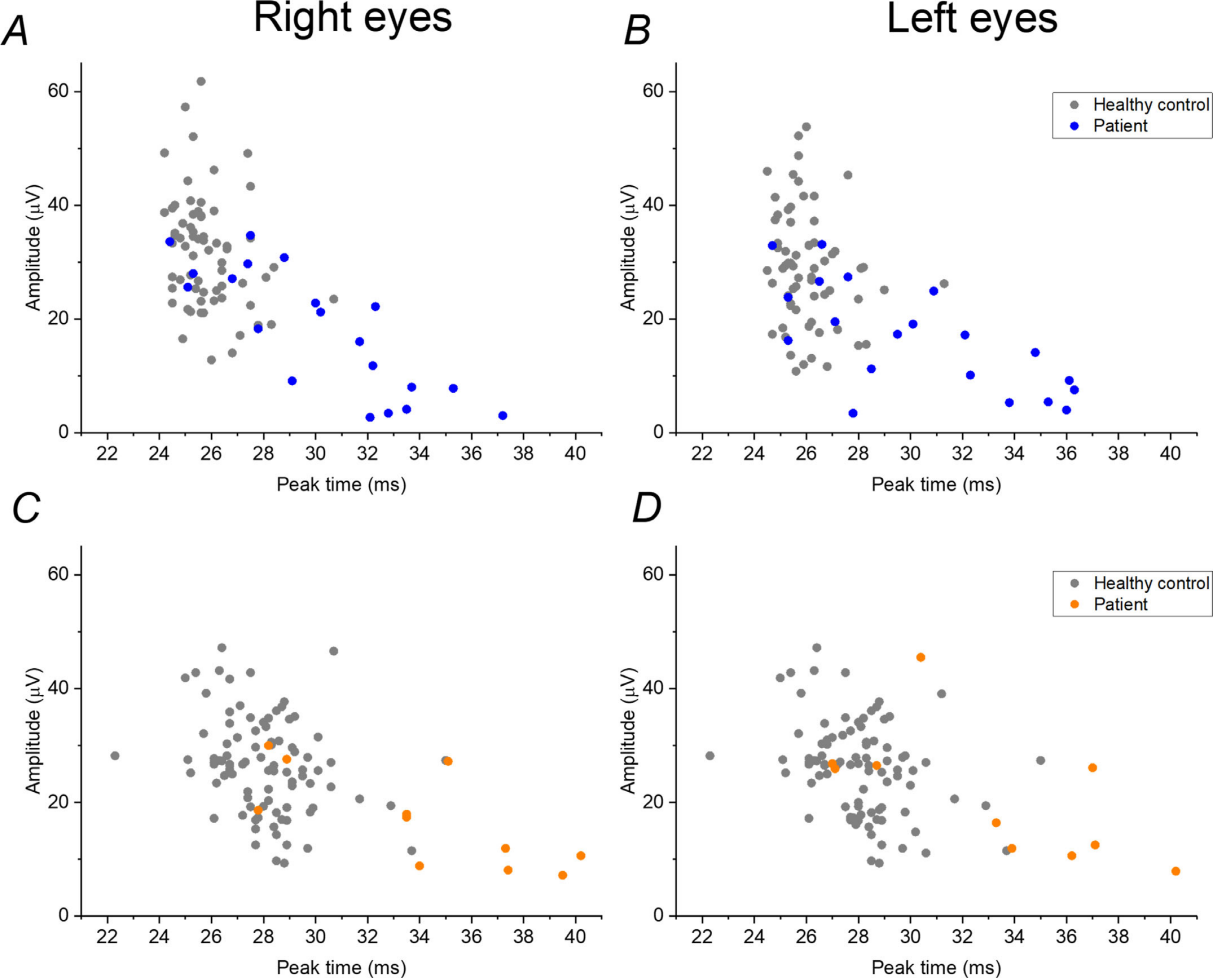
Amplitudes plotted against peak times for flicker ERGs recorded with skin electrodes according to the two protocols in healthy participants (*gray* *symbols*) and patients with BCR (*colored symbols*). (A, B) ERGs recorded in response to 28.3-Hz white flashes delivering 85 Td·s on an 850-Td white background. (C, D) ERGs recorded in response to 28.3-Hz white flashes delivering 32 Td·s (with no background).

Figure 3 plots amplitudes against peak times for a number of healthy controls (gray symbols), as well as the patients with BCR (colored symbols). The control recordings were obtained in healthy adults from the TwinsUK cohort (with only one twin per pair included). The upper panels show data from recordings made in response to the stimulus calculated to deliver retinal illuminance similar to the standard light-adapted 30-Hz flicker. These are a subset of data previously published.^11^ The mean age of that cohort was significantly younger than the patients with BCR; the cohorts were therefore age matched by removing some participants from the healthy cohort to give an age distribution similar to that of the BCR cohort (similar proportions per decade). The control data shown in the upper panels of Figure 3 are for 66 participants; the mean age of these participants was similar to that of the patients with BCR (*P =* 0.88).

The lower panels of Figure 3 plot data for recordings made in response to the 32-Td·s stimulus delivered with no background. The healthy controls shown are 90 healthy unrelated adults from the TwinsUK cohort (only one twin per pair included). The mean age of these participants was not significantly different from the patients with BCR (*P =* 0.11), so no further age matching was performed.

In both upper and lower panels, a general relationship is apparent whereby ERGs with longer peak times tend to have lower amplitudes. Many of the patients had markedly longer peak times than healthy subjects for both protocols. Patients tended to have lower amplitudes also; in group 1, several patients had lower amplitudes than the control participants. The Table gives the means for the data displayed in Figure 3, with results of comparisons between patients and control participants. Patients with BCR in both groups had significantly longer mean peak times and significantly lower mean amplitudes compared with healthy participants.

**Table.** Ages and ERG Parameter Values for Skin Electrode Recordings in Patients and Healthy Participants

		
		Mean (SD)		
			Healthy	*P* Value
		Patients	Participants	for Difference
Group 1 (85 Td·s, 850-Td background)				
Age (y)		55.8 (12.5)	55.3 (11.1)	0.88
ERG amplitude (µV)	Right eyes	17.2 (11.3)	31.9 (10.0)	<0.0001^*^
	Left eyes	15.7 (9.8)	29.1 (10.1)	<0.0001^*^
ERG peak time (ms)	Right eyes	30.2 (3.5)	25.8 (1.2)	<0.0001^*^
	Left eyes	30.3 (4.0)	26.0 (1.2)	<0.0001^*^
Group 2 (32 Td·s)				
Age (y)		57.5 (8.9)	64.5 (18.4)	0.11
ERG amplitude (µV)	Right eyes	16.8 (8.4)	26.6 (8.5)	0.0005^*^
	Left eyes	20.4 (11.0)	26.1 (8.3)	0.0404^*^
ERG peak time (ms)	Right eyes	34.1 (4.4)	28.0 (1.9)	<0.0001^*^
	Left eyes	33.9 (5.1)	28.1 (1.9)	<0.0001^*^

* *P* < 0.05 denotes significance.

## Discussion

Due to the progressive and variable nature of BCR and potential side-effects of treatment, objective methods of assessing disease activity are particularly important for guiding treatment. Fluorescein and indocyanine green angiography can yield valuable information; however, these investigations are invasive and, like clinical fundal examination, require pupil dilation. Electroretinography provides an objective, non-invasive assessment of retinal function, but this investigation is also time consuming and requires pupil dilation and specialist expertise, including in the placement of electrodes in the eye. Of the range of ERG parameters, the light-adapted 30-Hz flicker peak time appears in multiple reports to be a particularly sensitive measure.^2^^,^^4^^,^^5^^,^^14^ In this study, we explored using a portable device with specialized skin electrodes to record flicker ERGs without the need for pupil dilation, and we found a strong correlation with parameters from conventional recording methods.

The device was quick and convenient to use in the clinic or office setting, with the recording sessions typically taking less than 5 minutes and patients tolerating the electrodes and stimuli well. A number of flicker ERG protocols can be employed with the portable device. The non-mydriatic stimulus protocol based on the ISCEV standard delivers a retinal illuminance of 85 Td·s on a white background of 850 Td. In our study, this stimulus was used in 21 patients (group 1), and the amplitudes and peak times correlated very strongly with conventional recordings. A more widely available protocol on all versions of the RETeval device is a weaker flickering stimulus at the same frequency, but with no background, developed as part of a test to aid diabetic retinopathy screening. In the present study, this stimulus was used in 11 patients (group 2), and we also found strong correlation with conventional recordings to standard stimuli.

Amplitudes were significantly lower with the portable device, as expected with the use of skin electrodes. It has been shown previously that amplitudes are highly dependent on the position of the sensor strip skin electrodes,^11^ but this can also be the case with conventional conductive fiber electrodes.^15^ The smaller amplitudes obtained with the skin electrodes might mean that, in advanced disease, the recordings are less likely to detect reliable responses. Indeed, in two patients in our study, the ERG recorded in one eye with the portable device was too small to give a measurable peak time, although a peak time was measurable with conventional recordings. Amplitudes were on average approximately 3 to 3.5 times higher with conventional recordings in group 1 and approximately 4.5 to 5 times higher with conventional recordings in group 2. The difference in ratios is likely to partly reflect differences in methods of conventional recordings (gold foil or conductive fiber electrodes),^16^ as well as differences in stimulus characteristics (the portable device stimulus protocol in group 2 delivered a lower retinal illuminance). Given the variability in ratios in our patients, as well as the likely variability in stimulus and recording parameters in different laboratories, including electrode type and position,^11^^,^^15^^,^^16^ we would caution against deriving a universal scaling factor from our study; use of the same technique in longitudinal recordings is advisable.

Peak times appear to be less dependent on position of the sensor strips,^11^ and these may be a more sensitive marker of retinal dysfunction than amplitudes. Bland–Altman analysis assesses agreement between two quantitative methods of measurement (Fig. 2). Such analysis showed closer limits of agreement (with conventional recordings), as might be expected, for the flicker protocol that was closer to the ISCEV standard (group 1). Interestingly, this protocol gave statistically significantly shorter peak times on average than with conventional recordings, but the difference was very small (1.1 ms and 1.2 ms for right and left eyes, respectively). The other flicker protocol (group 2) gave longer peak times (by an average of 1.8 or 2.1 ms, respectively), and the difference was close to statistical significance. For longitudinal monitoring of patients, as discussed above, it would be advisable to use consistent testing methods. Our findings indicate that the ISCEV standard-equivalent protocol gives times very close to those obtained with conventional recording methods. These results suggest that, when this protocol is available, it should be selected for monitoring.

Significant and strong correlations were found between ERG parameters and time since diagnosis in group 1, but not in group 2. As expected, amplitudes correlated negatively with time since diagnosis (smaller amplitudes being recorded later in disease), and peak times correlated positively with time since diagnosis (increasing delay observed later in disease). The magnitude of the correlations appeared greater for amplitudes than peak times. One possibility is that amplitudes reflect the overall area of functioning retina, which would be expected to decline with disease duration, whereas peak times reflect the level of current (potentially reversible) disease activity, which might fluctuate with time. The focus of the present study was to compare portable with conventional recordings rather than to assess the utility of ERGs in monitoring disease (this has already been previously shown); the close similarity in correlation coefficients obtained with conventional and portable recordings would support the notion that they provide comparable assessments. The lack of significant correlations between ERG parameters and time since diagnosis in group 2 might reflect the smaller number of patients in this group, as well as the shorter duration (and range of durations) since diagnosis, compared with group 1.

We also compared parameters from portable recordings with the two protocols with those obtained in healthy individuals. Although there was some overlap as expected (because some patients will have early or quiescent disease), there were a number of patients with lower amplitudes and longer peak times compared with healthy controls. The separation in peak time was evident for both protocols, but the separation in amplitudes was more apparent for group 1 than group 2 patients. Mean amplitudes were significantly lower and mean peak times were significantly longer in patients compared with control participants, consistent with this being associated with the disease.

There are a number of limitations of the present study. Although the sample size is reasonable for such a rare disease, the small numbers preclude a formal comparison between the two portable device protocols, particularly as the two groups had different mean durations of disease. Future studies employing both protocols in the same patients would be of interest. That our study yielded similar results in separate analyses of right and left eyes supports the overall reproducibility of our main findings. We did not investigate longitudinal changes in ERG parameters. Given the prolonged disease course of BCR, future studies could aim to assess data longitudinally to explore whether changes in peak times and amplitudes over the course of disease and treatment are discernible using the handheld device. Usefulness of handheld ERG parameters in monitoring disease progression and response to treatment could also be explored by formal comparison with other investigations, such as optical coherence tomography, perimetry, fluorescein and indocyanine green angiography, and subjective visual function.

Also, although the photopic flicker peak time is regarded as the most sensitive parameter in BCR, other ERGs, including scotopic responses, are also affected.^2^^,^^4^^,^^5^^,^^17^^–^^20^ Some studies have suggested that analysis of oscillatory potentials from dark-adapted ERG or multifocal ERG parameters could be more sensitive. The electrooculogram at baseline has also been correlated with long-term outcome.^21^ Such abnormalities will be missed in purely photopic full-field testing.^17^^,^^18^ One possible approach would be the use of portable recordings to identify patients who might then undergo more extended recordings.

Our findings are likely to be applicable to other diseases in which panretinal cone-driven ERG responses are affected. Given the challenges in objectively diagnosing^22^ and managing BCR and the usefulness of photopic ERGs in monitoring disease activity, we were particularly interested in the applicability of portable recordings to this condition and hence undertook recordings specifically in patients with this diagnosis. Clinical decision-making to undertake an electroretinogram can be variable, depending on several factors including availability of the service and response to therapy. The opportunity to provide an in-clinic electroretinogram could expand the accessibility for this objective test. Future studies are likely to show applicability in other diseases.

## Conclusions

Our findings suggest that the portable ERG device has the potential to meaningfully evaluate patients with BCR in clinics where conventional ERG monitoring is not available or relatively inaccessible. Further, longitudinal studies will be helpful. Our results suggest that portable recordings could potentially be used in the office setting to provide a rapid assessment of generalized cone system function in these patients and might be applicable to other retinal diseases.
